# Understanding the Link between Animal Cruelty and Family Violence: The Bioecological Systems Model

**DOI:** 10.3390/ijerph17093116

**Published:** 2020-04-30

**Authors:** Brinda Jegatheesan, Marie-Jose Enders-Slegers, Elizabeth Ormerod, Paula Boyden

**Affiliations:** 1 Faculty of Educational Psychology, Learning Sciences and Human Development, University of Washington, 322 F Miller Hall, Box 353600, Seattle, WA 98195-3600, USA; 2 Faculty of Psychology, Open University of The Netherlands, Valkenburgerweg 177, 6419 AT Heerlen, The Netherlands; Marie-Jose.Enders@ou.nl; 3 Society for Companion Animal Studies (SCAS), P.O. Box 23, Chertsey, Surrey KT16 9WQ, UK; elizabeth.ormerod@btinternet.com; 4 Dogs Trust, 17 Wakely Street, London EC1V 7RQ, UK; Paula.Boyden@dogstrust.org.uk

**Keywords:** Bronfenbrenner’s bioecological systems model, animal abuse, animal cruelty, child abuse, family violence, the Link

## Abstract

Violence towards animals and violence towards people are often interconnected problems, and as such, this phenomenon has been termed the Link. Violence towards animals is a strong predictor that the abuser may inflict violence on people. However, it must not be assumed this is always the case. Professionals treating an animal or a human patient/client who has been subjected to abuse are uniquely situated to act in the role of ‘first responders’ when they suspect or recognize animal abuse, human abuse, or family violence. To more fully understand the Link the authors introduce Bronfenbrenner’s bioecological systems model through which to examine the complexity of the problem. Using data from earlier studies in which they interviewed police officers, other law enforcers, veterinarians, social workers, and community and family members, the authors discuss the correlation between animal cruelty and family violence. Furthermore, they examine how Bronfenbrenner’s bioecological systems model has the potential to better support animal and human health and welfare professionals in the identification of strategies for animals and humans caught in abusive settings. The authors recommend that these professionals become familiar with the bioecological systems model, which will enable them to better understand the psychological problems of animal cruelty and family violence and the different bioecological contributing factors. The authors emphasize transdisciplinary collaboration as vital in the recognition, prevention, and protection of animal and human victims trapped in family violence.

## 1. Introduction

### 1.1. Companion Animals in the Family System

Companion animals are increasingly becoming an integral part of family ecology worldwide. The number of households in the United States having a pet was estimated to be 67% [1]. In Europe, e.g., in The Netherlands, 59% of the households in France, 50% of the households and in the U.K., 40% of the households have companion animals [2,3,4]. A vast majority of these families consider their pet to be a family member and have deep emotional relationships with them. Research has demonstrated that many health, physical, psychological, and social benefits accrue from having companion animals across the life cycle [5,6].

Children and adolescents, particularly those who are vulnerable, derive significant benefits across a range of developmental areas from having a companion animal in their midst. These include social and emotional, cognitive, educational, and behavioral areas of positive influence [7,8,9,10,11,12,13]. Among adults, companion animals also serve as important sources of psychological and social support (e.g., provide comfort, reduce feelings of loneliness during stressful times, increase social connectedness and self-esteem) and improved physical health arising from increased exercise [14]. Studies have found that for children who frequently experience trauma, their companion animals become their confidants, comforters, and bosom buddies who help them heal emotionally, find solace, provide a sense of security, and relieve them of stress [8,15].

The primary goal of this paper is to introduce the bioecological systems model and propose that professionals addressing and/or treating an animal or a human being who have been subjected to abuse are uniquely situated to act in the role of ‘first responders’ when they suspect or recognize animal abuse, human abuse, and family violence. The authors introduce the bioecological systems model because they believe that this will help professionals understand the complexity of the problem. The model will also provide an insight into the different ways professionals from a range of disciplines can work together for guiding mental health prevention and interventions [16].

Four cases identified during clinical and research fieldwork of the authors are used to discuss the usefulness of the Bronfenbrenner bioecological model to illustrate the correlation between animal abuse and family violence [17,18,19,20]. The cases are in agreement with the findings of researchers worldwide who found correlations between the abuse directed towards partners, children, and companion animals [21,22,23,24].

### 1.2. One Health-One Welfare

The human–animal bond is recognized as a key aspect in both the One Health [25] and the One Welfare approaches. In the One Health and One Welfare approaches, factors that concern each area—humans, animals, and environment—are all considered [26]. One Health is not a new concept but dates from the introduction of veterinary training. Many of its applications involve veterinary and other scientists collaborating to protect public health. The One Health approach recognizes that the “health of the people is connected to the health of animals and the environment” and the “goal is to attain optimal health outcomes recognizing the interconnection between people, animals, plants and their shared environment” [26]. The Centers for Disease Control (CDC) defines health as “a state of complete physical, mental, and social wellbeing” [27]. CDC’s reference to emotional, social, and natural states can also be found in the definition of One Welfare which emphasizes the strong Link between animal welfare and human health [28]. In addition, the World Health Organization defines health as a state of complete physical, mental, and social wellbeing and not merely the absence of disease or infirmity. Jordan and Lem explain that “where there are poor states of human welfare there commonly exists poor states of animal welfare… Similarly, animals often act as indicators of human health and welfare as can be seen in the Link between animal abuse and family violence” [26] (p. 1203). More recently the transdisciplinary approach has been extended to One Health One Welfare [26,29].

### 1.3. The Link—Animal Cruelty and Family Violence

For companion animals, their status as family members comes with both benefits and possible harm. In most families, companion animals are an integral part of the family and are treated similarly to the rest of the family members (e.g., including playtime and walks, food and medical care, birthday celebrations, and sleeping together). However, in other families, they are subjected to the harshest and most unkind conditions of neglect, emotional and physical abuse [30]. Ascione defined animal cruelty as “socially unacceptable behavior that intentionally causes unnecessary pain, suffering, or distress to and/or the death of an animal [31] (p. 228). Cruelty to animals is also described as a multidimensional construct that includes among others, severity, duration, frequency, and lack of empathy [32,33], as well as physical and mental dimensions of cruelty [34]. The lack of standardized definitions of animal cruelty, types of animals involved, and the time frame within which the abuse occurred have been viewed as methodological shortcomings when it concerns reporting animal cruelty incidents [34,35].

#### 1.3.1. Animal Cruelty as a Marker

In a 2011 literature review conducted about animal abuse in the context of other violent and antisocial behaviors Gullone concluded that animal abuse is a marker of other potentially sinister experiences in children’s lives and that the relationship between animal abuse and aggression in childhood can extend into adulthood [36]. Often, companion animals can become victims during family violence, or used as pawns by the perpetrators to instill and enforce fear and control over their partner and children, creating interlocking systems of companion animal abuse, child abuse, and family violence [37,38,39,40,41,42,43,44,45].

The connection between the treatment of animals being closely associated with the treatment of fellow human beings was first documented in the 13th century [46]. Understanding of family violence and its repercussions have deepened over the past 50 years. The Battered Child Syndrome, a landmark paper in 1962 by Kempe et al., described the types of injuries received from deliberate physical abuse, usually perpetrated by a family member or a babysitter [47]. Following the publication of the Battered Child Syndrome there was initial reluctance in the medial professions to accept the evidence and act in such cases to prevent further abuse. Writing in 1964, Mead found that across a range of cultures, extraordinary abuse of animals (e.g., torture, killing) by children may precede more violent acts by that individual as an adult [48]. She argued that an act of cruelty towards an animal by a child could “prove a diagnostic sign, and that such children, diagnosed early, could be helped instead of being allowed to embark on a long career of episodic violence and murder (p. 22). Her writings influenced the American Psychiatric Association (APA) to add animal cruelty to the Diagnostic and Statistical Manual of Mental Disorders–III R (DSM-III R) in 1987. In the 2013 Diagnostic and Statistical Manual of Mental Disorders (DSM 5) animal cruelty was retained as a symptom of conduct disorder [49,50].

Additional studies in the 60′s also documented the Link between violence towards animals in childhood and aggressive behavior towards humans in adulthood [51,52]. Fucini, in 1978, made the Link between animal abuse and child abuse and expressed the belief that a battered pet may be indicative of other types of violence happening in the family [53]. Van Leeuwen, a child psychiatrist, writing in 1981 stated among so-called accidental injuries brought to veterinarian attention “It would be sad if in analogy to child abuse there persisted a reluctance to recognize the existence of animal abuse among the so-called accidental injuries brought to the veterinarian’s attention. Greater awareness of animal abuse may lead veterinarians to initiate mental health intervention for the abusing family in addition to treating the animal” [54] (p. 182). Indeed, there has been resistance to accepting the evidence that family violence frequently involves family pets. In 1981, Hutton, a social worker in England, highlighted that animal abuse could be used as a diagnostic indicator for family violence [55]. His study found that 82% of families known to the Royal Society for the Prevention of Cruelty to Animals (RSPCA) for animal abuse or neglect were also known to Social Services as having children at risk or having signs of physical abuse or neglect. He found clients were more willing to talk about their ill treatment of their pets, and from this, he could make better judgements as to when children were at risk. In 1983, in the International Journal for the Study of Animal Problems, DeViney et al. described a survey of pet owning families in the US with substantiated child abuse and neglect [56]. The authors found that animals were abused in 88 percent of homes in which children had been physically abused. In the majority of these cases, the abuse of the animals was perpetrated by a parent(s). The association between substantial animal directed abuse in childhood and later aggression towards people was confirmed by Kellert and Felthous in a 1985 paper [57]. In 1998, in a study published in the Journal of Emotional Abuse of abused women who sought shelter at a safe home and who had companion animals, 71 percent confirmed that their partner had threatened, injured or killed their pets [58]. Numerous studies in the millennium to date have documented the co-occurrence of family violence and animal abuse internationally [59,60,61,62,63,64].

#### 1.3.2. Impact of Witnessing Animal Cruelty

There is growing evidence that many children and adults have often witnessed one or more forms of family violence as well as animal cruelty [40,65,66,67]. Children and adults can be exposed to direct forms of abuse or they may indirectly have experienced the effects of abuse by virtue of being a witness of family violence that has included a companion animal. Companion animals are often considered as a cherished family member and can also be subjected to abuse as a form of intimidation and retaliation to have/maintain control and power by the perpetrator. Although all cases of family violence can have a negative effect on children, frequency, length, and severity of the violent act will influence its effect [68,69]. Children who are frequently exposed to severe forms of family violence are found more likely to often abuse animals as are children who are regularly exposed to animal abuse [70]. Both these groups of children are more inclined to repeatedly abuse animals as compared to children who have witnessed a few incidents of animal abuse [71]. Both direct and indirect forms of abuse have profound short and long term impact on child development. Five key areas of child development documented as having significant traumatic impact are physical or biological functioning, behavioral, emotional development, social adaptation, and cognitive development [72]. Children who witness or experience animal cruelty or neglect are more likely in the future to engage in the abuse of other animals and people, often mimicking the behavior of the perpetrator [73,74]. Children and adults who have a strong bond with their companion animals have been documented to intervene during incidents of abuse of their animals [75]. Such preventative acts of protection occur either verbally (e.g., pleading with the abuser) or physically (e.g., blocking their companion animals with their body).

In all cases of animal abuse, there is, besides the physical animal abuse, a psychological impact on the bystanders including the animals [54,76]. Victims who often choose to remain in family violence situations feel that they are unable to leave for fear of repercussions by the perpetrator on the companion animal [67,77]. Few family violence shelters accept companion animals, even though it is now well recognized this is why victims with animals delay fleeing and thus remain in jeopardy [45,58,77]. To address this problem some countries have introduced pet fostering services. The U.K. has a number of pet fostering services, e.g., Dogs Trust Freedom project which will house the companion animal while the victim is rehomed in a safe place [78]. A pet fostering service has recently been established in The Netherlands [79]. In addition, pilot programs in which companion animals stay together with their families in refuges have been recently introduced [80]. However, in most countries pet fostering services are still unavailable and there are very few refuges accepting companion animals from violent situations. This results in ongoing abuse and fatalities of women, children and animals that are preventable.

### 1.4. Bronfenbrenner’s Bioecological Systems Model

The Bronfenbrenner’s bioecological systems model introduced in this article emphasizes that the contexts in which an active developing individual spends time and the relations of the individual with others in the same setting, the personality of the individual (and those with whom he/she interacts), both in terms of development over time and the historical time in which these individuals live and the mechanisms that propel development (proximal process) should be considered [81]. Bronfenbrenner’s model has been a landmark advance in understanding family violence and in helping early detection of abuse and early intervention. The model has greatly advanced practitioners’ understanding of and response to family violence. It has been found to be a useful tool across health services and as such strongly recommended to be employed by organizations such as World Health Organization, Centers for Disease Control, and the United Nations (UN).

The bioecological systems model is characterized by four systems: microsystem, mesosystem, exosystem, and macrosystem. These systems enable the examination of human development within nested contexts of close relationships with individuals within the family (e.g., parents, siblings, companion animals), schools, neighborhoods, religious centers, health institutions, human health and safety services, animal welfare services among others. Socio-cultural beliefs and norms are also considered as influencing factors of human development. Interactions that occur between the systems are as much of importance and influence to human development as those interactions that occur within them.

The innermost circle and the closest layer to the individual is the microsystem, the individual’s immediate physical, social, and psychological environment. It consists of patterns of activities, social, and familial roles, and interpersonal relations with which he/she interacts in bidirectional or face to face settings (e.g., settings such as family, school, workplace, peer group). The second level, which is a system of microsystems, is known as the mesosystem. The mesosystem consists of interconnections and processes of two or more microsystem settings that involve the developing individual (e.g., relationship between home and school, home and workplace). Surrounding the mesosystem is the exosystem which comprises of interconnections and processes that happen between two or more immediate settings, of which in at least one setting the individual is not present or does not participate within it. However, events in this setting indirectly influence processes in the immediate environment in which the individual lives (e.g., for the child, the relationship between school and parent’s workplace) and from time to time the events are influenced by the individual. The macrosystem contains cultural beliefs, societal norms, socio-political factors, economic facts and government systems that affect the circumstances and processes existing in the microsystem. The hallmark of the macrosystem is its overarching belief system or ideology. It is the broadest ecosystemic level within which the microsystem and exosystem operate. The fifth and final system is the chronosystem which reflects change or continuity that occurs over the individual’s lifetime caused by events or experiences [82,83,84,85,86,87,88,89]. The critical message of this model is that all systems interact with and influence each other and are reflected in the development processes of the individual. It suggests that every small intervention in a system or between the systems will affect the micro system of the individual (and the companion animals) with potential to achieve positive or negative changes.

### 1.5. Animal Abuse and Family Violence: Integrating the Bioecological Systems Model

The multi-tiered set of systems illustrates the range of professionals who may be involved with the individual in his/her development process within and across the range of ecological contexts. In cases of family violence in which animal and human victims are evident, collaborative efforts can therefore be established between a range of relevant individuals and professionals for more effective interventions to be applied.

**Figure 1:**
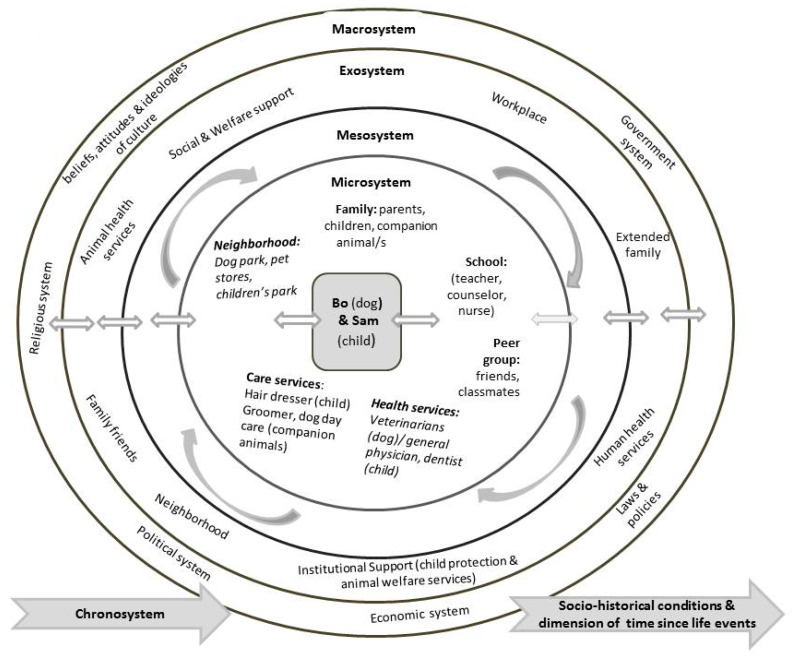
Bioecological Map.

With the ecological map in mind, the animal or human health and welfare professionals would begin the process of building knowledge by first examining the immediate environment (microsystem) of the subject human or companion animal. Understanding the whole context and making home visits are vital. Home visits allow for a better assessment of both human and animal welfare [90]. For example, using the ecological map of the hypothetical case of Bo and Sam in Figure 1, the concerned health and welfare professional can further his/her knowledge by simultaneously examining the microsystem of both, the companion animal (Bo) and the child (Sam), thus identifying the structures and individuals at the closest layer to both of them in which interpersonal relationships and interactions occur on a frequent basis. Should the animal health and welfare officer have concerns about the welfare of Bo then he/she could consider the quality of Bo’s life with his family, his interactions with his caregivers (Smith parents) and with Sam, the emotional reaction of the dog towards the Smith parents (e.g., does Bo, during the clinical visit, seek support/comfort from his caregiver or is he afraid and avoids physical contact) and vice-versa (e.g., are the caregivers aloof, unconcerned, disinterested about Bo during the clinical visit?). However, it has been documented that some abused dogs try hard to please their perpetrator, and appear to be bonded; and some perpetrators feign concern about animals they have abused. If during a consultation the professional suspects this may be a case of animal abuse he/she should not confront the caregiver. Occasionally careful questioning may be warranted, for example, when there are injuries that may be the result of a deliberate act. The concerned professional should aim to have the animal admitted for tests/treatment or observation. Admitting the animal allows an animal health professional (e.g., veterinarian) time to conduct a more detailed examination including checking for bruising, and also provides the time needed to undertake careful analysis of all available records in the clinic. The veterinarian should also check on the history of Bo’s prior visits in the clinic; and the records of any other animals in the family. Concerns should be discussed with other veterinarians in the practice, especially with those who have treated Bo or the family’s other companion animals. Veterinary technicians, nurses, and reception staff should be asked about any discrepancies in histories provided and their impressions arising from interface with the family. Similarly, such a procedure could be undertaken by a child or adult protection services professional when investigating the welfare of a child or an adult in the family. Where suspicions are raised, both animal and human welfare officers can compare their findings since it is known that where animals are at risk, people are often at risk and vice versa. Neighbors, friends and teachers, for example, can also be inquired about the welfare of the child in the concerned family.

The mesosystem in Figure 1 demonstrates the myriad ways in which individuals in the microsystem of Bo and Sam both separately and together are interconnected. These interconnections allow animal and human health and welfare professionals to attain additional information. They also demonstrate the need for follow up actions towards identification and prevention of abuse and neglect including providing support for the victims such as removal to a safe place and/or counseling. An examination of Bo and Sam’s exosystem identifies potential risks to both. These could include negative influences from peer groups or family friends who support aggressive behavior of the Smith parents, stress from loss of job, separation from extended family, which reduces additional family protection and support for Bo and Sam and a lack of institutional support (child protection and animal protection services). Any or all of the above may be associated with features of family violence. The macrosystem provides information on deep seated cultural beliefs on the acceptability of violent and aggressive behaviors towards animals, children, or an adult and a lack of government services or absence of policies or laws for their protection. Examples of chronosystem information that can assist human health and welfare professionals would be a history of life events over time, (e.g., perpetrator or a victim of abuse), changes in family structure through displacement or relocation, illness, and death all of which predict negative psychological and behavioral outcomes.

The model has the ability to systematically document the scope of the problem and add depth to the understanding of both positive and negative relationships between the companion animal and his/her family members and vice versa, as well as the many aspects of the animal and human environment and how they interact with and impact each other. Animal abuse, child abuse, family violence, and actions of prevention and intervention of abuse can be better understood and planned through this model.

### 1.6. Transdisciplinary Collaboration: An Approach for the Identification and Prevention of Violence and Protection of Animal and Human Victims

By situating the animal and the humans at the center of the model, we are able to examine the multiplicity of factors such as the family, community, institutions (e.g., schools, workplace), society, cultural factors, and historical events that can aggravate or lessen incidents of violence and subsequently the harmful effects on the animal and the human.

Historically, it has been animal welfare professionals who have been most attentive to and concerned about animal abuse in situations of family violence. However, recent times have seen an interdisciplinary interest among mental health and law enforcement professionals in the connection between cruelty to animals and family violence. Their interest has been attributed to the inclusion of animal cruelty into the American Psychiatric Association diagnostic criteria for conduct disorder in 1994 [91], several other important awareness campaigns and activities in the United States, and elsewhere in the world such as in The Netherlands, Sweden, United Kingdom, and France [92,93,94,95]. Furthermore, overwhelming evidence on the Link between cruelty to animals and violence against children and adults in family settings across a range of disciplines (judiciary, police, psychology, social work, veterinary sciences, medicine, nursing) have further substantiated the occurrences [67].

Intervention, crucial in the identification, prevention, and protection of victims of family violence requires transdisciplinary collaboration. Psychologists, social workers, veterinarians, educators and other health and behavioral professionals witness an amalgam of victims/clients whose lives have been tragically impacted by family violence. The complex and fragile nature of this problem cannot be solved using any single discipline nor can it be addressed through the actions of an individual professional [96]. On the contrary, for a change in outcome, every effort must be made by a range of professionals to stand unified in sharing responsibility and working towards a common goal, each depending on the expertize of the other. Flexibility, contribution towards, and collective ownership of goals, interdependence, and maximizing the expertize of one another are all viewed as hallmarks of successful interdisciplinary collaboration [96,97,98,99].

Animal health and welfare professionals have traditionally worked independently of the other health and social care professions. When presented with cases of suspected animal abuse and/or family violence they face difficult challenges in emotionally charged situations, many of which would benefit from a multidisciplinary approach.

## 2. Methodology

In this article, the authors introduced the bioecological theoretical model of Bronfenbrenner. To demonstrate the model the authors provided cases that were selected from four separate studies conducted in The Netherlands, United States, and the United Kingdom [17,18,19,20]. The studies used one or more qualitative methods such as interviews, observations, video, and document analysis (e.g., family violence records, incident reports, duty logs). The participants were police officers, other law enforcers, veterinarians, social workers, community, and family members. The cases were selected from the studies to serve as examples of how the bioecological model could have helped reveal the abuse to the professionals involved. The authors incorporated the cases into the bioecological model of Bronfenbrenner to reflect the usefulness of this model in cases of family violence and animal cruelty.

### Ethical Issues

Formal ethics approval of the cases was obtained from the Institutional Review Board at the University of Washington, Seattle, WA, USA (IRB ID: STUDY00003277). Consent from the interviewees was attained. A pseudonym was assigned to each individual (humans and animal) to protect his or her identity. Interviews were conducted in the participants’ preferred language.

## 3. The Link between Child Abuse, Animal Abuse, and Family Violence: Four Cases of Connections

Four cases were purposefully selected as exemplars because of their relevance to the phenomenon of the Link between violence towards animals and humans and how it is intricately related to the behavior of individuals in each case and to demonstrate that understanding these cases requires a wide sweep of familial, historical, social, and cultural contexts. Furthermore, the cases provide an opportunity to learn about the particularity and complexity of the contexts in which the Link occurs. Additionally, the cases illustrate how the bioecological model has the potential to support animal and human health, welfare, and protection professionals in the identification and follow up strategies to prevent further abuse on animals and humans caught in abusive settings. Case 4 also demonstrates the value of professionals working together with transdisciplinary colleagues and institutions (e.g., social workers, animal welfare services) and organizations (e.g., companion animal foster services, refuges for human and animal victims of abuse) that extend beyond one’s usual practice in their respective fields in order to aid in the psychological and physical wellbeing of their patients and clients.

Each of the four cases presented below is accompanied by a case synopsis, contextual information, case analysis using the bioecological model, and an ecological map to demonstrate the Link between animal cruelty and child abuse in family violence.

### 3.1. Case 1 Synopsis: The Abuse of Sisters Ena and Mai and Their Dog Bamboo (USA)

Chan often threatened his two daughters Ena and Mai to comply with his orders by yelling at their dog Bamboo until she cowered in fear. There were times when Chan hit and kicked Bamboo in front of Ena and Mai until Bamboo cried and Ena and Mai would tearfully plead with their father to stop hurting Bamboo and promised that they would do anything he wanted them to do. Their mother Kiku remained as a silent witness to the frequent incidents of abuse of her daughters and their family dog taking no action to protect them. Kiku attributed her silence to socio-cultural, familial issues and social isolation. Attempts by Kiku’s friend to convince her to get help remained unfruitful.

#### 3.1.1. Case 1: Contextual Information

Sisters Ena and Mai lived with their mother (Kiku) and father (Chan) both first generation immigrant parents. Bamboo is their family dog and Ena and Mai are deeply bonded with her. The sisters’ best friend is Micki who is their classmate and neighbor. Micki’s mother, Yuna, is a friend of Kiku. Both families are of the same cultural background. Chan frequently emotionally and physically abused his daughters and their family dog. The sisters were forced to watch the abuse of Bamboo as a way to control them. During this time Ena and Mai would try to shield their dog and emotionally implore their father to show mercy on Bamboo and promised to comply with his wishes. It became clear to the sisters that their mother, who was often present when the abuse occurred, was helpless to intervene and end the trauma. The sisters considered informing their teacher Ms. Sasha but were hesitant because they viewed her as unfriendly and not welcoming of informal interactions with students. Instead, the sisters turned to their friend Micki and shared with her the abuse inflicted on them and Bamboo. Micki, distraught at what she heard disclosed their situation with her mother.

Yuna’s attempts to convince Kiku to get help were in vain. Kiku felt that family violence was not unusual in their community and that it was quite natural for husbands to have outbursts since they had stressors in their lives as a result of being the primary providers in the family. Kiku, who comes from a traditional family was unwilling to report her husband’s abuse for fear of shaming him in the eyes of her extended family and the community. She felt that it was her role to protect the family honor. Additionally, she believed in adhering to gender-specific responsibilities such as silent sacrifice, unquestioning loyalty, and complete obedience to male authority which is expected in a patriarchal family system. She also felt that there was not much she could do because of her dependency on her husband, limited English proficiency, and her lack of support of extended family members because they lived a distance away in her native country. Yuna discussed Kiku’s situation with a psychologist whom she had known for a few years and was given advice on next steps and offered assistance. When the psychologist followed up with Yuna to check if she provided Kiku with the information, Yuna uncomfortably responded ‘not yet.’ Numerous requests to Yuna by the psychologist for an update including offers of help for the victims failed. Yuna eventually stopped visiting the psychologist and multiple attempts to contact her were unsuccessful.

#### 3.1.2. Case 1: Analysis Using the Bioecological Systems Theory

**Figure 2:**
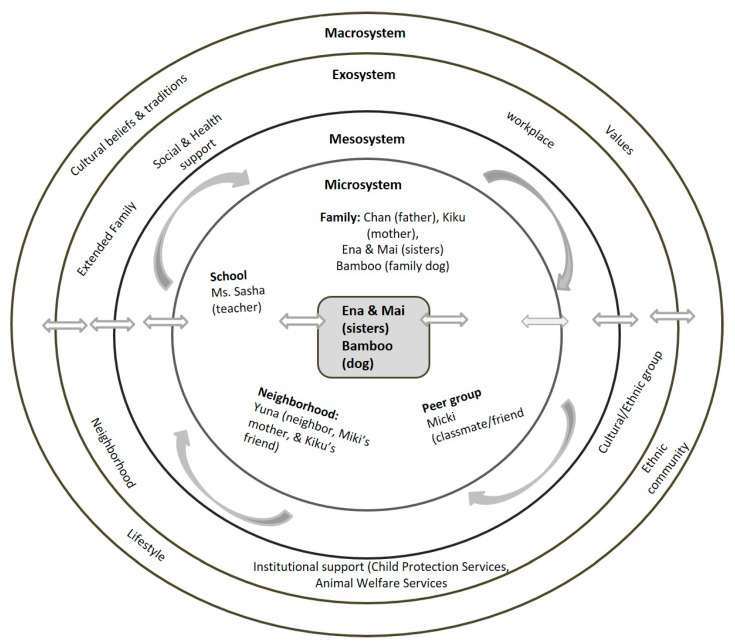
Case 1 Bioecological Map.

As shown in Figure 2 individuals within the microsystem of Ena and Mai and Bamboo are the parents/caregivers (Chan and Kiku), a school friend Micki and her mother Yuna and their teacher Ms. Sasha. The sisters experienced significant trauma in witnessing the abuse of Bamboo by their father. During this time they took emotional (i.e., pleading) and physical (shielding) actions to intervene and protect Bamboo from their father. Children’s interventions in preventing violence towards their companion animals and the protective strategies they have utilized have been reported in DeGue, et al. (2011) and Jegatheesan (2013) [15,18]. The sisters chose to confide in Micki (microsystem) about the abuse their father inflicts on them and on their dog Bamboo. Kiku’s explanations to Yuna for not taking her advice on reporting the abuse revolves around a lack of structures in her exosystem such as social and institutional support and absence of extended family members. Macrosystem factors associated with her unwillingness to report her husband’s violent behavior stem from traditional cultural beliefs, such as bringing shame to the family if she went public about her husband’s abusive behavior and through seeking help. As a result she chose to remain silent and in doing so felt she was safeguarding family honor. It is also critical to note that although Kiku harbors a strong cultural sentiment of acceptance and tolerance of abuse she nevertheless identified barriers such as a high level of dependency on her husband, limited English proficiency, and absence of familial and cultural support system in the USA, all of which could come in the way of her seeking assistance. It is important to note that the barriers identified by Kiku increased her social isolation and which proved to be a significant challenge in her getting connected to institutions of support such as child protection services, animal welfare services, and law enforcement (exosystem). Finally, this case illustrates a situation where the plight of the victims may have been improved had the family friend implemented the steps advised by the professional she consulted. Numerous studies of immigrant women have demonstrated that social isolation is strongly related to a higher risk of family violence [100,101,102].

### 3.2. Case 2 Synopsis: The Killing of an Infant in a Family (UK)

An animal protection agency inspector visited a house in February due to reports by neighbors of much yelling. When he arrived, Coco, a 6-month-old puppy, belonging to the young couple Brad and Kate was found to be lame with a bruised eye. Kate reported that Coco had ‘fallen’ down the stairs when her partner Brad beat him for chewing the suitcase which was packed ready for her to go into hospital to give birth. Brad was upset and said that ‘his head was all over the place’. Coco was signed over to the animal protection agency. Brad thought this best due to the imminent birth of the baby. Baby Kyle was born the following month. In May of the same year Brad was charged with his young son Kyle’s murder. Initially, Kate said that the baby had ‘fallen’ down the stairs. Brad later admitted to throwing his young son down the stairs.

#### 3.2.1. Case 2: Contextual Information

An infant boy tragically died when his father (Brad) threw him down the stairs in this case of family violence. Brad’s abusive and violent nature was established 3 months prior to the murder of his infant son when he beat Coco, the family’s puppy over a chewing incident. An officer from an animal protection agency who visited the house to investigate the situation determined that the puppy had been subjected to abuse and Coco was relinquished to the animal protection agency. The animal protection agency conducts private prosecutions, which do not require mandatory reporting to the law enforcement agency. What remains unknown in this case is whether Brad had previously abused Coco, or if he was violent towards his wife Kate. 

#### 3.2.2. Case 2: Analysis Using the Bioecological Systems Theory

**Figure 3:**
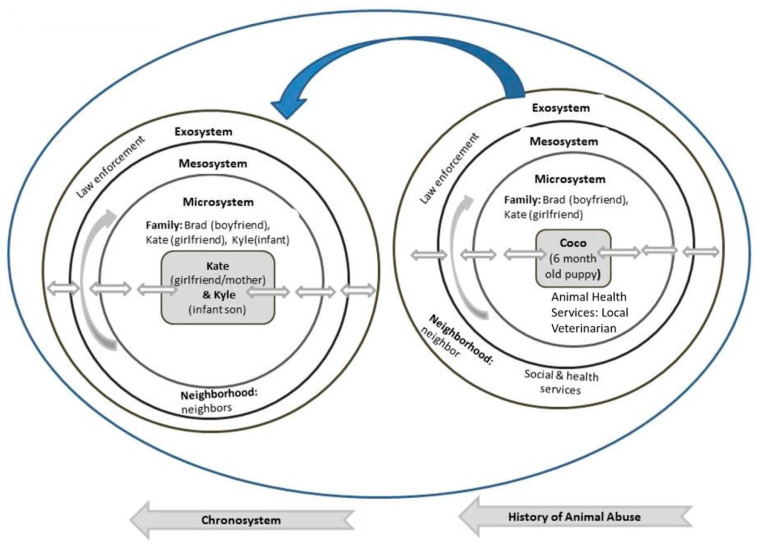
Case 2 Bioecological Map.

As illustrated in Figure 3 individuals within the microsystem of Coco the puppy and baby Kyle are parents Brad and Kate and their neighbors. Brad’s abusive and violent nature was established 3 months prior to the murder of his infant son when he beat Coco, the family’s puppy over a chewing incident. Neighbors concerned by the sounds of screaming and yelling called the animal protection agency (exosystem). Although an animal protection officer (exosystem) came to the house, and determined that the puppy had been abused, it is unclear if a report was made to the human health organization in view of the fact that Kate was heavily pregnant. Often it has been found that animal abuse has been treated as an isolated incident and not deserving of a police report [103,104]. Furthermore, some investigators are not yet aware of the interconnectedness between child abuse, animal abuse, and family violence and of the need for cross-reporting.

The fact that Brad’s abusive behavior was established, an early report to social services could have provided Brad with an opportunity to get early intervention and support such as counselling and/or anger management to help avert recurring abusive behavior. At that time, there was no evidence that Brad was violent towards Kate. This case is a typical example of a scenario where violence towards animals could have been used as a significant indicator for later family violence [105,106,107]. Such scenarios should prompt attending professionals to alert appropriate agencies, in this case, the social services and mental health agencies (exosystem). 

Awareness of the Link could have prompted several actions. These include reporting the abuse to the police for their input, separately interviewing the pregnant woman about her situation and that of the puppy, talking with neighbors to obtain additional information as well as seeking information from Coco’s veterinarian. The information gathered could have provided clearer insights about the actual situation, and subsequently alerted social services and other relevant services in providing support to the victims and to the perpetrator. 

It is important to consider the very short time interval between the abuse of Coco and baby Kyle’s death. Even if cross-reporting was undertaken there would have been insufficient time for effective intervention. This case demonstrates the correlation between the incident of Brad abusing Coco and the murder of his infant 3 months later (chronosystem).

### 3.3. Case 3 Synopsis: A Lack of Timely Intervention in a Troubled Youth Who Brutally Killed a Kitten and Continued Violence a Decade Later (The Netherlands)

A police officer was called by neighbors to an apartment where a young father named Tim had beaten Jill, his ex-wife, and threatened to beat their baby as well. Tim had been violent before and was legally forbidden to come near Jill’s house. The police officer recognized Tim as one of the youngsters in a mall ten years ago who had kicked a male kitten and thrown him against a wall to his death. At the time of the kitten’s abuse, the police reported the incident to Tim’s parents in his presence. Tim’s parents were not concerned with what he had done; instead, they consoled him with a reward.

#### 3.3.1. Case 3: Contextual Information

This case describes how a police officer (Bob) recognizes a young man (Tim) from a previous violent situation. The first time that Tim was confronted by the police was when he was fourteen years old. On that occasion, Bob was the police officer on duty and was called to a mall where a group of 14- to 16-year-old boys had been kicking a kitten. Witnesses reported that the kitten had stroked their legs, which irritated them. When the kitten did not stop looking for attention one of the boys (Tim) took the kitten into an alley, where he smashed the kitten several times against the wall. An older lady tried to stop the abuse, picked up the dying kitten, and called the police. Bob went with his colleague to meet Tim’s parents to report the incident. He stated that he “never forgot the reaction of Tim’s mother and father.” Tim’s parents were not concerned about what Tim had done and gave him money to buy an ice cream as a means of consolation. The police officers did not inform Social Services (which is *now* an obligation in The Netherlands).

Ten years later, a police officer was called by neighbors of a young mother (Jill), whose ex-husband Tim had come to her house and beaten her. Tim also threatened to beat their infant named Ned. Jill had divorced Tim because of his severe violence towards her. Tim was convicted for his violence towards his wife and legally forbidden to come near the house. Coincidentally the police officer who responded to the incident was Bob. Bob recognized Tim and looked through his files for earlier cases of violence. The abuse and killing of the kitten in the mall was filed in Tim’s police record.

#### 3.3.2. Case 3: Analysis Using the Bioecological Systems Theory

**Figure 4:**
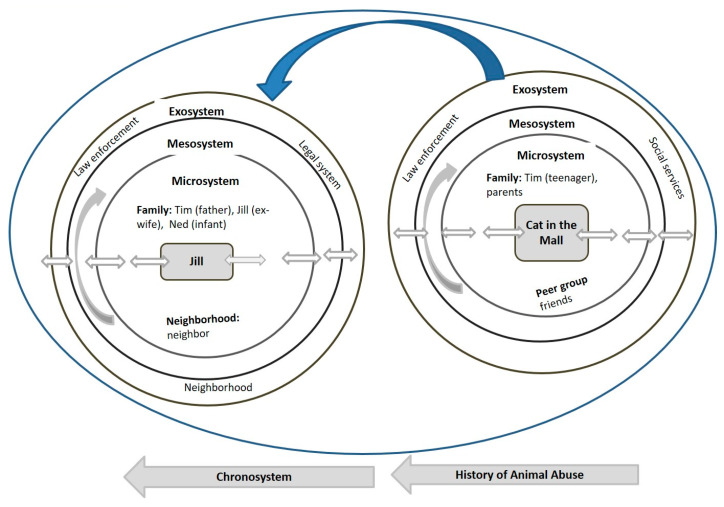
Case 3 Bioecological Map.

As illustrated in Figure 4 individuals within the microsystem of Tim as a child were his parents and his friends. Tim along with his friends was involved in a merciless act of violence when he abused and brutally killed a kitten in a mall, that was witnessed and reported by an older lady (exosystem). When viewed through the ecological systems lens, several individuals in Tim’s ecological system failed to take appropriate action for his egregious act of cruelty. These included the investigating police officer Bob (exosystem) who did not file charges against Tim and did not report the killing of the cat to animal welfare services (exosystem). Furthermore, Bob did not inform social and health services (exosystem), which resulted in Tim not being provided with timely and appropriate interventions to help prevent violence in the future. Studies have shown that violence towards animals during childhood can be a warning sign of future abusive behavior as an adult [108,109].

In addition, Tim’s family was not investigated about possible family violence. Other individuals who failed to act appropriately when Tim mercilessly killed the kitten were his parents (microsystem) who did not reprimand him or seek social and health support services (exosystem) for him such as mental health evaluation, diagnosis, and targeted counselling. Tim’s brutality towards a kitten years prior can be viewed as a strong predictor of later violence at an older age and his actions causing extreme stressors and effects on the victims. The lack of cross-reporting by the police officer and Tim’s parents, regarding the specific incident in the mall, was a missed opportunity for timely intervention. Targeted support during his adolescence (chronosystem) may have prevented the escalation of his abusive behavior as occurred in his violent relationship with Jill, his ex-wife, and their infant a decade later (adult microsystem).

Traditionally, animal abuse by children has been examined as a separate issue [110]. Because animal abuse cases may reveal a wider spectrum of behaviors related to the Link, the authors make a compelling argument for the need for cross-reporting as a way to inform the various expressions of violence to their colleagues in other disciplines. Viewed as best practice, cross-reporting requires coordinated and collaborative interdisciplinary efforts with information sharing among animal and child welfare agencies, social and human services agencies, law enforcement departments, and other related disciplines concerned with family violence.

### 3.4. Case 4 Synopsis: Woman and Dog, Who Had Both Been Subjected to Years of Abuse, Rescued by a Veterinarian and Her Transdisciplinary Team (UK)

A veterinarian on emergency duty, was called to a house early one morning to attend to a dog in pain. The woman named Sarah had been awoken by her dog named Kerry screaming. Her husband who had been downstairs with her dog told her that Kerry had fallen off the settee. Sarah was disheveled and very distressed whilst her husband was smartly groomed and aloof. Something fell from Sarah-a urinary collection bag. As the veterinarian returned this she experienced a “light bulb” moment. Could this be a case of family violence against Sarah and Kerry? The husband left the room briefly and the veterinarian quickly passed her private phone number to Sarah, mouthing “phone me”.

#### 3.4.1. Case 4: Contextual Information

Sarah was seriously physically abused by her husband Blake over many years. Her doctor advised her to leave Blake but offered her no support or referral and did not report the abuse to the police. Sarah had also been frequently admitted to hospital after being battered. Sarah lost one of her kidneys through the injuries and the other was seriously damaged. At the hospital, neither the attending doctors nor other hospital staff questioned Sarah about her injuries. Sarah had contacted local women’s refuges, but none would allow companion animals. Her dog Kerry was also repeatedly abused by Blake and had been presented to a local veterinarian on many occasions. He was puzzled by the case and had not been able to diagnose the cause of Kerry’s repeated pain episodes.

Early on a Sunday morning, an Out of Hours veterinarian who was on emergency duty for three veterinary practices answered Sarah’s call requesting a home visit for her dog. However, on questioning Sarah it did not sound like an emergency situation requiring a home visit since Kerry was already receiving ongoing veterinary treatment for pain. Furthermore, Sarah’s own veterinarian who had been attending to Kerry was available for consultation that morning, so the duty veterinarian suggested that Sarah wait until then. However, Sarah pleaded with her to attend to Kerry and as there was such desperation in her voice the duty veterinarian agreed to visit. Their home was in another town, some miles away.

On arrival at the house, Blake answered the door and led the way to the living room where the veterinarian found Kerry lying on the floor and Sarah sitting on the settee looking very unkempt. Kerry was in discomfort but was receiving the appropriate veterinary medication at the correct dose. The veterinarian felt her presence was neither needed nor warranted. However, as she knelt on the floor in front of the settee to examine Kerry more carefully Sarah provided more history. Sarah had been upstairs in bed when she heard Kerry screaming. Blake who had been downstairs with Kerry told Sarah that Kerry had fallen off the settee. The veterinarian determined that Kerry’s pain was sublumbar, in the renal area.

During the consultation, Blake stood against a wall observing and did not contribute to the consultation. Then something fell off the settee landing just behind the veterinarian who reached back without looking to pick it up and return it to Sarah, thinking this would be a magazine. To her astonishment, it was a urine collection bag. Then came a moment of revelation and things came together—Sarah’s illness, Kerry’s mystery illness, the repeated bouts of pain, the discrepancy between the couple’s attitude, behavior, and demeanor. The history of Kerry falling off the settee didn’t seem right, and only Blake was present when Kerry cried out. The fact that Kerry’s pain was in the sublumbar region and Sarah had a urinary tract problem. The veterinarian considered that this could be a case of family violence. She remained calm and gave no outward sign of her suspicions. She wrote her home number on her business card intending to slip it to Sarah before she left. However, a few minutes later Blake went into the kitchen and the veterinarian passed her card to Sarah whilst silently mouthing “phone me”.

After leaving Sarah’s house the veterinarian, to check if her intuitions were accurate, discussed Sarah’s circumstances with one of her colleagues, a social worker who specializes in family violence. The social worker stated that this was a classic presentation of a family violence situation and advised her to alert the client’s veterinarian of her suspicions. When she called Kerry’s veterinarian later that morning he could not accept that Blake could be battering the dog. Kerry’s veterinarian said Blake was a “nice and caring man who always accompanied his wife to the surgery and who expressed great concern about the dog”.

Later that afternoon Sarah phoned the veterinarian who had visited her home out of hours and talked at length about her abusive situation. She disclosed that Blake was a violent man who spent years in prison for acts of violence where he had undertaken anger management classes in prison. Despite the ongoing abuse and her grave medical condition Sarah was in conflict about leaving her violent husband. She stated that she was a Christian and “had taken her marriage vows.” She felt bound to remain with her husband. She saw no way out of her situation, except death. Sarah made no reference to friends or family. The veterinarian told Sarah that Kerry hadn’t taken any such vows, that she had a duty of care for Kerry and that if she did not leave Blake would likely kill both Kerry and her. She informed Sarah that she would contact Paws for Kids an organization that would place Kerry in a loving foster home until they were reunited.

This veterinarian had created a transdisciplinary community network as part of her bond-centered veterinary practice. With the help of this veterinarian and her transdisciplinary team Sarah agreed to get help. Paws for Kids was contacted and the organization placed Kerry with a foster caregiver. Sarah was admitted to a safe house. A month later this veterinarian received a letter from Sarah expressing heartfelt thanks and stating she would soon be reunited with Kerry in a new home.

#### 3.4.2. Case 4: Analysis Using the Bioecological Systems Theory

**Figure 5:**
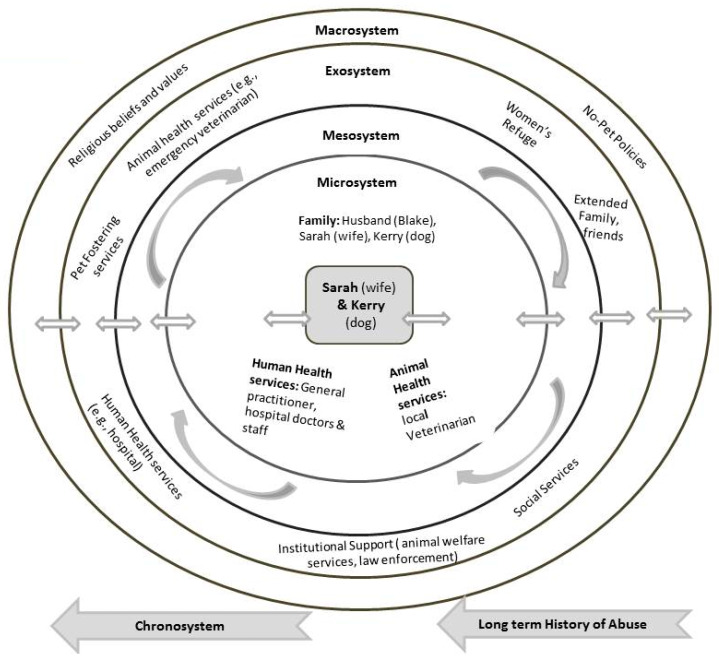
Case 4 Bioecological Map.

As demonstrated in Figure 5, individuals and interactions with them in Sarah’s immediate surroundings are her husband Blake and her dog Kerry (microsystem). Sarah was severely battered by Blake over many years. Throughout this time Sarah was in repeated contact with her local physician and with medical staff in a hospital for treatment of her life-threatening injuries. She was also in contact with her veterinarian for treatment of Kerry’s recurrent and unexplained pain (microsystem). Furthermore, Sarah also contacted local women’s refuges (exosystem) but their ‘no-companion animals’ policies meant that she could not take Kerry with her, and so Sarah decided to remain at home for fear of Kerry’s safety. Sarah’s decision to remain in her dangerous situation is consistent with results from previous research about battered women’s concerns for their companion animals’ safety affecting their decision about leaving for a family violence refuge or remaining in an abusive relationship [44,58,111]. This situation calls for local refuges to consider allowing companion animals on their premises as a matter of urgency.

The above-mentioned professionals in Sarah’s micro and exosystems, with whom she had interacted with during the course of her abuse, could have played a key role in eliminating the one component (viz. husband) in her “system” by informing the police and ending the trauma for her and Kerry. Informing the police would have led to uncovering the knowledge that Blake had a prior police record for acts of violence, had been imprisoned for many years for violence against another individual, and had undertaken anger management sessions in prison (chronosystem of Blake). These professionals could also have provided resources and services that could have been instrumental in helping Sarah and Kerry escape the abuse and move to a violence-free environment. Additionally, had the family veterinarian been aware of the Link, he too could have put an end to Kerry and Sarah’s trauma by seeking human and animal welfare services for the dyad. Intake policies in refuges that are inclusive of companion animals could have altered Sarah’s decision to remain in her abusive home situation with Blake, leaving them both vulnerable to abuse. An additional factor that played a role in Sarah’s hesitation to leave her husband was her religious belief (macrosystem) about her marriage vows. The factors indicated above collectively left Sarah and Kerry in continuous peril. Lastly, the chronosystem of the dyad illustrates the non-normative events in Sarah and Kerry’s life such as the abuse they endured over time and the lack of actions by professionals during the period of trauma that significantly impacted their prospects of a violence-free life. Sarah and Kerry’s situation remained unchanged until the duty veterinarian visited. She had, a priori, conceived and formed a transdisciplinary network of colleagues from other health and social care professions and organizations [19,112], and this proved to be pivotal in helping Sarah and Kerry leave their abusive situation.

## 4. Conclusions

This article introduces the Bronfenbrenner’s bioecological systems model (1973–2006) to understand the complex psychological problems of animal abuse, child abuse, and family violence, and its potential to aid professionals in the identification of strategies for animals and humans caught in family violence situations.

The catastrophic effect of violence to animals and violence to people underscores the need for transdisciplinary and collaborative interventions between animal welfare agencies and human services organizations to help lives trapped in family violence. A large and growing body of scientific research demonstrates the correlation that can exist between animal abuse, child abuse, and family violence, otherwise known as the Link. Therefore, animal and human agencies must work together to ensure integrated, thorough, and effective interventions. The old way of working in silos within one’s profession and using piecemeal approaches has clearly demonstrated its limitations and ineffectiveness as evident in cases 2, 3, and 4 in this article. The bioecological systems model offers more comprehensive effective ways to understand the complexity of animal cruelty and family violence.

Shared training is one way of helping professionals to understand their respective roles and responsibilities, build relationships, and gain insight into each other’s points of view and decision-making processes. The experience of learning from each other and learning together in both formal and informal ways can be a good basis for building trust and respect, empathy being one of the key interpersonal skills that support successful relationships and collaborative efforts [113].

The benefits to animal and human health and welfare professionals of being a member of a transdisciplinary network will become fully apparent when such complex cases arise and professionals are encouraged to keep the Bronfenbrenner model in mind. By alerting the appropriate agencies to cases of suspected abuse of their clients these professionals can play an important role in client protection and in enhancing their safety. Greater protection from violence and neglect would then be provided to children, women, vulnerable elders, and animals and preventive measures can be taken.

## References

[1] American Pet Products Association 2019–2020. https://www.americanpetproducts.org/pubs_survey.asp.

[2] Report Feiten en Cijfers Gezelschapdierensector 2015. https://www.rijksoverheid.nl/documenten/rapporten/2015/11/03/feiten-cijfers-gezelschapsdierensector-2015.

[3] Notre Planet. https://www.notre-planete.info/actualites/3751-nombre-animaux-compagnie-France.

[4] Pet Food Manufacturers Association Annual Report 2019. https://www.pfma.org.uk/_assets/docs/annual-reports/PFMA-2019-Annual-Report.pdf.

[5] McNicolas J., Gilbey A., Rennie A., Ahmedzai S.H., Dono J.A., Ormerod E. (2005). Pet ownership and human health: A brief review of evidence and issues. Br. Med. J..

[6] Podberscek A., Serpell J. (2000). Companion Animals and Us.

[7] Geerdts M. (2015). (Un)Real animals: Anthropomorphism and early learning about animals. Child Dev. Perspect..

[8] Jegatheesan B. (2009). The give and take in the Human-Animal Bond: Three tales of spirit healing. Reflect. Narrat. Prof. Health.

[9] Jegatheesan B., Meadan H. (2010). Pets in the Classroom: Promoting and Enhancing the Socio-Emotional Wellness of Young Children. Young Except. Child. Monogr..

[10] McNicolas J., Collis G.M. (2001). Children’s representations of pets in their social networks. Child Care Health Dev..

[11] Melson G., Peet S., Sparks C. (1991). Children’s attachment to their pets: Links to socio-emotional development. Child. Environ. Q..

[12] Melson G. (2003). Child development and the human companion animal bond. Am. Behav. Sci..

[13] Levinson B., Mallon G. (1997). Pet-Orientated Child Psychotherapy.

[14] McConnel A.R., Brown C., Martin C.E. (2011). Friends with benefits: On the positive consequences of pet ownership. J. Personal. Soc. Psychol..

[15] DeGue S., DiLillo D. (2009). Is animal cruelty a “red flag” for family violence? Investigating co-occurring violence toward children, partners, and pets. J. Interpers. Violence.

[16] Richard L., Gauvin L., Raine K. (2011). Ecological models revisited. Their uses and evolution in health promotion over two decades. Annu. Rev. Public Health.

[17] Garnier W., Enders-Slegers M.J. (2012). Huiselijk Geweld en Dierenmishandeling in Nederland. Een Verkennend Onderzoek naar de Relatie Tussen Huiselijk Geweld en Dierenmishandeling onder Vrouwelijke Slachtoffers van Huiselijk Geweld.

[18] Jegatheesan B. Muslim American children’s views on animal wellness and ethics. Proceedings of the International Association of Human-Animal Interaction Organizations (IAHAIO) Conference.

[19] Ormerod E.J. (2008). Bond-centered veterinary practice: Lessons for veterinary faculty and students. J. Vet. Med. Educ..

[20] Boyden P., Jegatheesan B. (2018). Personal communication.

[21] Ascione F.R., Weber C.V., Thompson T.M., Heath J., Maruyama M., Hayashi K. (2007). Battered pets and domestic violence: Animal abuse reported by women experiencing intimate violence and by nonabused women. Violence Against Women.

[22] Baldry A.C. (2003). Animal abuse and exposure to interparental violence in Italian youth. J. Interpers. Violence.

[23] McIntosh S.C. (2004). The Links between Animal Abuse and Family Violence, as Reported by Women Entering Shelters in Calgary Communities.

[24] Jury A., Thorburn N., Bury K., National Collective of Independent Women’s Refuges (2018). Pet Abuse as Part of Intimate Partner Violence. https://womensrefuge.org.nz/wp-content/uploads/2019/11/Pet-Abuse-Report-.pdf.

[25] WSAVA One Health Committee Report 2012. https://www.vin.com/apputil/content/defaultadv1.aspx?pId=11349&id=5328347.

[26] Jordan T., Lem M. (2014). One Health, One Welfare: Education in practice. Veterinary students’ experiences with community veterinary outreach. Can. Vet. J..

[27] The Centers for Disease Control and Prevention (CDC) One Health. https://www.cdc.gov/onehealth/index/html.

[28] Frasier D. (2009). Assessing animal welfare: Different philosophies, different scientific approaches. Zoo Biol..

[29] Pinillos R.G., Appleby M., Manteca X., Scott-Park F., Smith C., Velarde A. (2016). One Welfare—A platform for improving human and animal welfare. Vet. Rec..

[30] Newberry M. (2017). Pets in danger: Exploring the link between domestic violence and animal abuse. Aggress. Violent Behav..

[31] Ascione F.R. (1993). Children Who Are Cruel to Animals: A Review of Research and Implications for Developmental Psychopathology. Anthrozoos.

[32] Ascione F.R., Thompson T.M., Black T. (1997). Childhood cruelty to animals: Assessing cruelty dimensions and motivations. Anthrozoos.

[33] McPhedran S. (2009). Animal abuse, family violence, and child wellbeing: A review. J. Fam. Violence.

[34] Vermeulen H., Odendaal J.S.J. (1993). Proposed typology of companion animal abuse. Anthrozoos A Multidiscip J. Interact. People Anim..

[35] Connor M., Currie C., Lawrence A.B. (2018). Factors influencing the prevalence of animal cruelty during adolescence. J. Interpers. Violence.

[36] Gullone E. (2011). Conceptualizing animal abuse with an antisocial behavior of framework. Animals.

[37] Arluke A., Levin J., Luke C., Ascione F. (1999). The relationship of animal abuse to violence and other forms of antisocial behavior. J. Interpers. Violence.

[38] Ascione F.R., Ascione F.R., Arkow P. (1999). The abuse of animals and human interpersonal violence: Making the connection. Child Abuse, Domestic Violence, and Animal Abuse: Linking the Circles of Compassion for Prevention and Intervention.

[39] Ascione F.R., Weber C.V., Wood D.S. Animal Welfare and Domestic Violence Report Submitted to the Geraldine Dodge Foundation, April 1997. http://www.vachss.com/guest_dispatches/ascione_2.html.

[40] Barrett B.J., Fitzgerald A., Stevenson R., Cheung C.H. (2017). Animal Maltreatment as a Risk Marker of More Frequent and Severe Forms of Intimate Partner Violence. J. Interpers. Violence.

[41] Becker F., French L. (2004). Making the links: Child abuse, animal cruelty and domestic violence. Child Abus. Rev..

[42] Boat B.W. (1995). Commentary: The relationship between violence to children and violence to animals. J. Interpers. Violence.

[43] Flynn C.P. (2011). Examining the links between animal abuse and human violence. Crime Law Soc. Chang..

[44] Flynn C.P. (2000). Why Family Professionals Can No Longer Ignore Violence toward Animals. Fam. Relat..

[45] Flynn C.P. (2000). Woman’s best friend: Pet abuse and the role of companion animals in the lives of battered women. Violence Against Women.

[46] Regan T., Singer P. (1976). Animal Rights and Human Obligations.

[47] Kempe C.H., Silverman F.N., Steele B.F., Droegemueller W., Silver H.K. (1962). The battered child syndrome. J. Am. Med. Assoc..

[48] Mead M. (1964). Cultural factors in the cause and prevention of pathological homicide. Bull. Menn. Clin..

[49] American Psychiatric Association (1987). Diagnostic and Statistical Manual of Mental Disorders.

[50] American Psychiatric Association (2013). Diagnostic and Statistical Manual of Mental Disorders.

[51] McDonald J.M. (1963). The threat to kill. Am. J. Psychiatry.

[52] Hellman D.S., Blackman N. (1966). Enuresis, firesetting and crFuelty to animals: A triad predictive of adult crime. Am. J. Psychiatry.

[53] Fucini S. (1978). The abuser: First a dog then a child?. Am. Hum..

[54] Van Leeuwen J., Fogle B. (1987). A child psychiatrist’s perspective on children and their companion animals. Interrelations between People and Pets.

[55] Hutton J.S. Animal abuse as a diagnostic approach in social work: A pilot study. Proceedings of the International Conference on the Human-Companion Animal Bond.

[56] De Viney E., Dickert J., Lockwood R. (1983). The Care of Pets within Child Abusing Families. Int. J. Study Anim. Probl..

[57] Kellert S.R., Felthous A.R. (1985). Childhood cruelty toward animals among criminals and non-criminals. Hum. Relat..

[58] Ascione F.R. (1998). Battered women’s reports of their partners’ and their children’s cruelty to animals. J. Emot. Abus..

[59] Krienert J.L., Walsh J.A., Matthews K., McConkey K. (2012). Examining the nexus between domestic violence and animal abuse in a national sample of service providers. Violence Vict..

[60] Volant A.M., Johnson J.A., Gullone E., Coleman G.J. (2008). The Relationship between Domestic Violence and Animal Abuse: An Australian Study. J. Interpers. Violence.

[61] Ascione F.R. Men in Prison Who Abused Animals and Who Abused Their Wives and Girlfriends: Voices of Perpetrators. Proceedings of the 11th International Conference on Human-Animal Interactions, People & Animals: Partnership in Harmony (IAHAIO).

[62] Bright M.A., Huq M.S., Spencer T., Applebaum J.W., Hardt N. (2018). Animal cruelty as an indicator of family trauma: Using adverse childhood experiences to look beyond child abuse and domestic violence. Child Abuse Negl..

[63] Simmons C., Lehmann P. (2007). Exploring the link between pet abuse and controlling behaviors in violent relationships. J. Interpers. Violence.

[64] Faver C., Cavazos A. (2007). Animal abuse and domestic violence. A view from the border. J. Emot. Abus..

[65] Appel A.E., Holden G.W. (1998). The co-occurrence of spouse and physical child abuse: A review and appraisal. J. Fam. Psychol..

[66] Campbell A.M., Thompson S.L., Harris T.L., Wiehe S.E. (2018). Intimate partner violence and pet abuse: Responding law enforcement officers’ observations and victim reports from the scene. J. Interpers. Violence.

[67] Roguski M. (2012). Pets as Pawns: The Co-Existence of Animal Cruelty and Family Violence. Report for Royal New Zealand Society for the Prevention of Cruelty to Animals and the National Collective of Independent Women’s Refuges. http://nationallinkcoalition.org/wp-content/uploads/2013/01/DV-PetsAsPawnsNZ.pdf.

[68] Edleson J.L. (1999). Children’s witnessing of adult domestic violence. J. Interpers. Violence.

[69] Margolin G. (2005). Children’s exposure to violence: Exploring developmental pathways to diverse outcomes. J. Interpers. Violence.

[70] Currie C.L. (2006). Animal cruelty by children exposed to domestic violence. Child Abus. Negl..

[71] Thompson K.L., Gullone E. (2006). An investigation into the association between the witnessing of animal abuse and adolescents’ behavior towards animals. Soc. Anim..

[72] Kolko J.R., Blakely E.H., Engelman D. (1996). Children who witness domestic violence: A review of the empirical literature. J. Interpers. Violence.

[73] Parkes D., Signal T. (2017). Revisiting a link: Animal abuse, bullying, and empathy in Australian youth. Hum. Anim. Interact. Bull..

[74] Walters G. (2019). Animal cruelty and bullying: Behavioral markers of delinquency risk, or causal antecedents of delinquent behavior?. Int. J. Law Psychiatry.

[75] McDonald S.E., Collins E.A., Nicotera N., Hageman T.O., Ascione F.R., Williams J.H., Graham-Bermann S.A. (2015). Children’s experiences of companion animal maltreatment in households characterized by intimate partner violence. Child Abus. Negl..

[76] Arkow P. (1995). Breaking the Cycles of Violence: A Practical Guide.

[77] Faver C.A., Strand E.B. (2003). To leave or to stay?: Battered women’s concern for vulnerable pets. J. Interpers. Violence.

[78] Dogs Trust Freedom Project. https://www.dogstrust.org.uk/help-advice/hope-project-freedom-project/.

[79] Stay Away from My Animal. https://www.blijfvanmijndier.nl.

[80] The Orange House. https://www.blijfgroep.nl.

[81] Bronfenbrenner U., Richardson F. (1973). Social ecology of human development. Brain and Intelligence: The Ecology of Child Development.

[82] Bronfenbrenner U. (1974). Developmental research, public policy, and the ecology of childhood. Child Dev..

[83] Bronfenbrenner U. (1975). Reality and research in the ecology of human development. Proc. Am. Philos. Soc..

[84] Bronfenbrenner U. (1977). Toward an experimental ecology of human development. Am. Psychol..

[85] Bronfenbrenner U. (1979). The Ecology of Human Development.

[86] Bronfenbrenner U., Bolger N., Capsi A., Downy G., Moorehouse M. (1988). Interacting systems in human development. Research paradigms: Present and future. Persons in Context: Developmental Processes.

[87] Bronfenbrenner U. (1994). Ecological models of human development. International Encyclopedia of Education.

[88] Bronfenbrenner U., Freidman S.L., Wachs T.D. (1999). Environments in developmental perspective: Theoretical and operational models. Measuring Environment across the Life Span: Emerging Methods and Concepts.

[89] Bronfenbrenner U., Morris P.A., Damon W., Lerner R.M. (2006). The bioecological model of human development. Handbook of Child Psychology.

[90] Ormerod E. Animal-assisted Interventions: Animal welfare and the role of the veterinarian. Proceedings of the American Veterinary Medical Association (AVMA) Congress, International Association of Human-Animal Interaction Organizations (IAHAIO) Conference.

[91] American Psychiatric Association (1994). Diagnostic and Statistical Manual of Mental Disorders.

[92] Lockwood R. (2002). Making the connection between animal cruelty and abuse and neglect of vulnerable adults. Latham Lett..

[93] Enders-Slegers M.J., Janssen M.A. (2009). Cirkel van geWeld. Verbanden Tussen Dierenmishandeling en Huiselijk Geweld.

[94] Enders-Slegers M.J., Verheggen T., Jannes E., Pregowski M. (2009). Awareness can change a society: The Link between animal abuse and domestic violence in The Netherlands. Companion Animals in Everyday Life.

[95] Norden N. (2018). Experience in Sweden Making the LINK. One Health, One Welfare: The Importance of Research, Education and Cooperation.

[96] Bruner C. (1991). Ten Questions and Answers to Help Policy Makers Improve Children’s Services.

[97] Berg-Weger M., Schneider F.D. (1998). Interdisciplinary collaboration in social work education. J. Soc. Work Educ..

[98] Mattessich P., Monsey B., Amherst M. (1992). Collaboration: What Makes It Work.

[99] Soler M., Shauffer C. (1993). Fighting fragmentation: Coordination of services for children and families. Educ. Urban Soc..

[100] Abraham A. (2000). Isolation as a form of marital violence: The South Asian immigrant experience. J. Soc. Distress Homeless.

[101] Fagan J.A., Browne A., Reiss A.J., Roths J.A. (1994). Violence between spouses and intimates: Physical aggression between women and men in intimate relationships. Understanding and Preventing Violence.

[102] Gelles R.G. (1997). Intimate Violence in Families.

[103] Jegatheesan B., Enders-Slegers M.J., Arkow P., Boyden P. Examining the relationship between animal abuse and child abuse. Proceedings of the International Association of Human-Animal Interaction Organizations (IAHAIO) Conference.

[104] Jegatheesan B., Enders-Slegers M.J. The LINK between child abuse and animal abuse: A Challenge for veterinarians and strategies for collaborative partnerships for preventative, protective and healing interventions. Proceedings of the International Society for Anthroozoology (ISAZ) Conference.

[105] Munro H.M., Ascione F.R., Arkow P. (1999). The battered pet. Child Abuse, Domestic Violence, and Animal Abuse: Linking the Circles for Prevention and Intervention.

[106] Munro H.M., Thrusfield M.V. (2001). ‘Battered pets’: Features that raise suspicion of non-accidental injury. J. Small Anim. Pract..

[107] Lockwood R., Hodge R., Lockwood R., Ascione F. (1997). The tangled web of animal abuse: The Links between cruelty to animals and human violence. Cruelty to Animals and Interpersonal Violence.

[108] Felthous A.R., Kellert S.R. (1986). Violence against animals and people: Is aggression against living creatures generalized. Bull. Am. Acad. Psychiatry Law.

[109] Felthous A.R., Kellert S.R. (1987). Childhood cruelty and later aggression against people: A review. Am. J. Psychiatry.

[110] Ascione F.R., Arkow P. (1999). Child Abuse, Domestic Violence, and Animal Abuse: Linking the Circles of Compassion for Prevention and Intervention.

[111] Weber C.V. (1998). A Descriptive Study of the Relation between Domestic Violence and Pet Abuse. Ph.D. Thesis.

[112] Ormerod E.J. The Bond-centred veterinary practice: Strategies for supporting the human-companion animal bond within the veterinary surgery and the wider community. Proceedings of the People and Animals: Partnership in Harmony, the 11th International Conference on Human-Animal Interactions.

[113] Carpenter J., Szilassy E., Patsios D., Hackett S. (2010). Outcomes of Interagency Training to Safeguard Children: Final Report to the Department for Children, Schools and Families and the Department of Health.

